# Supplementary material for: Short-course radiotherapy combined with chemotherapy and PD-1 inhibitor in low-lying early rectal cancer: study protocol for a single-arm, multicentre, prospective, phase II trial (TORCH-E)
Source: BMJ Open. 2023 Oct 6;13(10):e076048. doi: 10.1136/bmjopen-2023-076048 (PMC10565143; doi:10.1136/bmjopen-2023-076048)
Supplement: Author's manuscript [file bmjopen-2023-076048.draft_revisions.pdf]

BMJ Open is committed to open peer review. As part of this commitment we make the peer review history of every article we publish publicly available.

When an article is published we post the peer reviewers' comments and the authors' responses online. We also post the versions of the paper that were used during peer review. These are the versions that the peer review comments apply to.

The versions of the paper that follow are the versions that were submitted during the peer review process. They are not the versions of record or the final published versions. They should not be cited or distributed as the published version of this manuscript.

BMJ Open is an open access journal and the full, final, typeset and author-corrected version of record of the manuscript is available on our site with no access controls, subscription charges or pay-per-view fees (http://bmjopen.bmj.com).

If you have any questions on BMJ Open's open peer review process please email info.bmjopen@bmj.com

BMJ Open

Short-course radiotherapy combined with chemotherapy and PD-1 inhibitor in low-lying early rectal cancer: a single-arm, multi-center, prospective, phase II trial (TORCH-E)

Journal:	*BMJ Open*
Manuscript ID	bmjopen-2023-076048
Article Type:	Protocol
Date Submitted by the Author:	26-May-2023

Complete List of Authors:	Chen, Yajie; Fudan University Shanghai Cancer Center, Department of Radiation Oncology<br>Wang, Yaqi; Fudan University Shanghai Cancer Center, Department of Radiation Oncology<br>Zhang, Hui; Fudan University Shanghai Cancer Center, Department of Radiation Oncology<br>Wan, Juefeng; Fudan University Shanghai Cancer Center, Department of Radiation Oncology<br>Shen, Lijun; Fudan University Shanghai Cancer Center, Department of Radiation Oncology<br>Wang, Yan; Fudan University Shanghai Cancer Center, Department of Radiation Oncology<br>Zhou, Menglong; Fudan University Shanghai Cancer Center, Department of Radiation Oncology<br>Wu, Ruiyan; Fudan University Shanghai Cancer Center, Department of Radiation Oncology<br>Yang, Wang; Fudan University Shanghai Cancer Center, Department of Radiation Oncology<br>Zhou, Shujuan; Fudan University Shanghai Cancer Center, Department of Radiation Oncology<br>Cai, Sanjun; Fudan University Shanghai, Department of Oncology; Fudan University Shanghai Cancer Center, Department of Colorectal Surgery<br>Li, Xinxiang; Fudan University Shanghai, Department of Oncology; Fudan University Shanghai Cancer Center, Department of Colorectal Surgery<br>Zhang, Zhen; Fudan University Shanghai Cancer Center, Department of Radiation Oncology<br>Xia, Fan; Fudan University Shanghai Cancer Center, Department of Radiation Oncology

Keywords:	GASTROENTEROLOGY, RADIOTHERAPY, ONCOLOGY

SCHOLARONE™ Manuscripts

Short-course radiotherapy combined with chemotherapy and PD-1 inhibitor in low-lying early rectal cancer: a single-arm, multi-center, prospective, phase II trial (TORCH-E)

Yajie Chen[1,2,3,4], Yaqi Wang[1,2,3,4], Hui Zhang[1,2,3,4], Juefeng Wan[1,2,3,4], Lijun Shen[1,2,3,4], Yan Wang[1,2,3,4], Menglong Zhou[1,2,3,4], Ruiyan Wu[1,2,3,4], Wang Yang[1,2,3,4], Shujuan Zhou[1,2,3,4], Sanjun Cai[2,5], Xinxiang Li[2,5*], Zhen Zhang[1,2,3,4*] and Fan Xia[1,2,3,4*]

1 Department of Radiation Oncology, Fudan University Shanghai Cancer Center, Shanghai, China.

2 Department of Oncology, Shanghai Medical College, Fudan University, Shanghai, China.

3 Shanghai Clinical Research Center for Radiation Oncology, Shanghai, China.

4 Shanghai Key Laboratory of Radiation Oncology, Shanghai, China.

5 Department of Colorectal Surgery, Fudan University Shanghai Cancer Center, Shanghai, China.

First author: Yajie Chen

***Corresponding author:**

Xinxiang Li, Department of Colorectal Surgery, Fudan University Shanghai Cancer Center, Shanghai, China. Email: xinxiangli@fudan.edu.cn

Zhen Zhang, Department of Radiation Oncology, Fudan University Shanghai Cancer Center, Shanghai, China. Email: zhen_zhang@fudan.edu.cn

Fan Xia, Department of Radiation Oncology, Fudan University Shanghai Cancer Center, Shanghai, China. Email: tcxiafan@hotmail.com

For peer review only

Abstract

Background: Current standard treatment for patients with early rectal cancer is radical surgical resection. Although radical surgery provides effective local tumour control, it also increases the mortality risk and considerable adverse effects, including bowel, bladder, sexual dysfunction and loss of anal function, especially in patients with low-lying rectal cancer. Recent studies have shown promising synergistic effects of the combination of immunotherapy (PD-1/PD-L1 inhibitors) and radiotherapy in improving tumour regression. For patients who reach a clinical complete response (cCR) after neoadjuvant therapy, a "Watch & Wait" (W&W) approach can be adpoted to preserve anorectal function and improve quality of life. Thus, this study aims to explore the efficacy and safety of radiotherapy combined with chemotherapy and PD-1 antibody in low early rectal cancer patients.

Methods: TORCH-E study is designed as a multi-center, prospective, phase II trial of short-course radiotherapy (SCRT) combined with chemotherapy and PD-1 inhibitor in cT1-3bN0M0 low rectal cancer patients. The enrolled 34 patients will first receive SCRT (25 Gy/5Fx), followed by four cycles of CAPOX chemotherapy and PD-1 antibody (Toripalimab), and finally receive surgery or the W&W strategy. The primary endpoint is the complete response (pCR plus cCR) rate. The secondary endpoints include the organ preservation rate, 3-year local recurrence free survival (LRFS) rate, 3-year disease free survival (DFS) rate, 3-year overall survival (OS) rate, grade 3-4 adverse effects rate, and patients' quality of life (QoL).

Discussion： The results of this trial will provide a novel insight into SCRT in combination with chemotherapy and Toripalimab in the treatment of early low rectal cancer patients, which is expected to provide new therapeutic option for patients who are pursuing the W&W strategy to preserve the rectum.

Trial registration: NCT05555888 (ClinicalTrials.gov); Date of registration: September 22, 2022; URL of trial registry record: https://clinicaltrials.gov/ct2/show/NCT05555888

Keywords: Early low rectal cancer, Immunotherapy, Short-course radiotherapy, Complete response, Organ preservation.

Strengths and limitations of this study:

1. This clinical trial is the first prospective, multi-center, phase II study using short-course radiotherapy combined with PD-1 inhibitor and chemotherapy in early low-lying rectal cancer.
2. This study is of great clinical significance for achieving organ preservation in patients with early low-lying grade rectal cancer.
3. This is a phase II clinical study involving a small number of patients. Further expansion of the sample size and conducting a phase III clinical trial will be necessary to validate the effectiveness and safety.

For peer review only

Background

The standard treatment for cT1-3bN0M0 (stage I-II) rectal cancer remains to be radical surgical resection. Other therapeutic approaches are currently in the exploratory stage, but provide a promising direction. To be specific, local excision (TEM) is curative in T1N0 rectal cancer without any pathological factors of poor prognosis. In stage T2 and T1 with poor prognosis factors, the standard treatment is total mesorectal excision (TME) due to the high recurrence rates recorded with TEM[1]. In terms of stage T3 rectal cancer, neoadjuvant chemoradiotherapy(nCRT) combined with TME surgery is the standard treatment. Although surgical resection provides effective local tumour control, it could increase the mortality risk and the incidence of considerable adverse effects, including high stoma rates, faecal incontinence, and urinary- and sexual dysfunction[2], especially in patients with low-lying rectal cancers. Most survivors will experience functional sequelae due to removal of the rectum, which significantly impairs their quality of life (QoL). With the increasing need of improved quality of life, organ preservation after reaching clinical complete response (cCR) via neoadjuvant therapy is gaining interest for rectal cancers.

It is generally known that neoadjuvant chemoradiotherapy (nCRT) plays a significant role in achieving organ preservation in locally advanced rectal cancer (LARC). Nowadays, with the optimization of the nCRT therapies, including enhancing the intensity of concurrent chemotherapy, increasing the interval of chemotherapy, and performing total neoadjuvant therapy (TNT) strategy, the complete response (CR) and organ preservation rate have been further improved[3, 4]. Patients who reach a complete clinical response after nCRT can adopt the "Watch & Wait" (W&W) strategy to preserve the rectum[5]. In addition, TNT model is also expected to reduce distant metastasis and improve long-term survival[6, 7]. Treatment goals for the neoadjuvant therapy of LARC have changed from reducing local recurrence to promoting tumour regression, increasing organ preservation, and improving long-term survival. As neoadjuvant therapy can significantly reduce the tumour stage, increase the sphincter preservation rate, decrease the rate of local recurrence, nCRT might have a promising role in early rectal cancer patients who have difficulty with anal sphincter preservation. In the past two years, a number of clinical studies on the exploration of chemoradiotherapy for early rectal cancer have emerged, such as the OPERA study, STAR-TREC phase II study, indicating that neoadjuvant radiotherapy and organ preservation strategies for early-stage rectal cancer have received increasing attention. These studies have shown good cCR rate and 3-year organ preservation rate, but the long-term efficacy is still being followed up.

The remarkable efficacy and survival benefit of immunotherapy (PD-1/PD-L1 inhibitors) have been widely demonstrated in the treatment of various tumours[8-12]. Emerging preclinical evidence suggested that the combination of radiotherapy with PD-1/PD-L1 antibody can modulate the tumour microenvironment, reverse the immunosuppressive effects, increase the secretion of T-cell-derived antitumour cytokines, and enhance the efficacy of radiotherapy [13, 14]. Recently, a number of clinical studies on nCRT combined with immunotherapy are ongoing. The combination of nCRT with immunotherapy has been reported to achieve outstanding short-term efficacy and satisfactory safety in LARC patients. These clinical trials are mainly divided into two modes: long-course radiotherapy (LCRT) and short-course radiotherapy (SCRT) combined with immunotherapy. The major clinical trials of the LCRT combined with PD-1/PD-L1 inhibitors included VOLTAGE-A, PANDORA and NSABPFR-2, etc.[15-17]. The CR (pCR + cCR) rates in these three studies all reached 30% or greater, which is much higher than the pCR rate of traditional nCRT (15-20%).

For peer review only

Besides, the treatment model of hypofractionated SCRT combined with PD-1/PD-L1 inhibitors is also commonly applicated. The TORCH study conducted by Fudan University adopted the TNT model, performed SCRT combined with 6 cycles of Toripalimab and CAPOX, and reported the preliminary results at the 2022 ASCO meeting[18, 19]. The results remarkably showed that 60.4% (29/48) patients achieved CR, 60.7% (17/28) achieved pCR，78.6% (22/28) reached MPR, and the organ preservation rate was 88.9% (40/45)[20]. In addition, another two SCRT-based trials also showed high pCR rates. The Averectal trial performed SCRT followed by mFOLFOX6 plus Avelumab for LARC patients[21]. The pCR rate was 37.5%, the near-pCR rate was 30%, and the MPR rate was 67.5%. Another study reported at the 2023 ASCO-GI Symposium found that neoadjuvant SCRT followed by Envafolimab plus CAPOX in LARC cancer could achieve a promising high pCR rate of 76.6%[22]. These prospective studies suggest that the treatment approach of CRT combined with chemotherapy and PD-1/PD-L1 monoclonal antibody for LARC patients is expected to make higher tumor regression and CR rate compared to traditional chemoradiotherapy. Thus, it is reasonable to infer that this strategy is likely to achieve satisfactory tumor response and organ-preservation rate when applied to low-lying early rectal cancer.

Based upon the above findings and our previous TORCH study, the treatment approach of combining PD-1 monotherapy with SCRT and chemotherapy is expected to remarkably improve tumour regression and CR rate, and provide more options for patients with early rectal cancer to achieve organ preservation. Moreover, about 95% of patients with rectal cancre have the proficient mismatch repair (pMMR)/ microsatellite-stable (MSS) type, which is not sensitive to PD-1 inhibitor alone. Therefore, we are conducting a phase II trial of the combination of short-course radiotherapy, CAPOX and Toripalimab for neoadjuvant therapy in pMMR/MSS, early and low rectal cancer, with the expectation to explore the efficacy and safety of this combination therapy.

Methods

Study design

The TORCH-E study is a multi-center, prospective phase II trial of short-term radiotherapy combined with chemotherapy and PD-1 inhibitor in early low rectal cancer. The enrolled patients will receive short-term radiotherapy (25 Gy/5Fx), followed by 4 cycles of CAPOX chemotherapy and PD-1 antibody (Toripalimab), and finally receive TME surgery or local excision (TEM). The complete response (pCR plus cCR) rate, organ preservation rate, long-term prognosis, and adverse effects will be analyzed. The scheme of study progress was showed in Fig. 1.

Trial organization, Ethics, and informed consent

This trial is investigator initiated by the Department of Radiation Oncology, Fudan University Shanghai Cancer Center (FUSCC). Five other cancer centers in China are involved in the study, including Renji Hospital, Shanghai Changzheng Hospital, Fujian Cancer Hospital, the Second Affiliated Hospital Zhejiang University School of Medicine and the Second Affiliated Hospital of Soochow University. Before enrollment of first patient, the study protocol was approved by the Ethics Committee of all these research centers, and all patients provided written informed consent before recruitment. Shanghai Junshi Biomedical Technology Co., Ltd provides PD-1 antibody Toripalimab free of charge and purchases liability insurance for clinical trial subjects.

Patient and public involvement

Neither patients nor the public will be involved in the design, recruitment, assessment and conduct of this study. The results will be disseminated via peer-reviewed scientific journals and conference presentations rather than specifically notified to a single patient.

Study population

Inclusion in the TORCH-E trial requires that certain tumour and patient criteria are met. The inclusion and exclusion criteria are presented in Table 1.

Table 1 Inclusion and exclusion criteria

Inclusion criteria	Exclusion Criteria
1. Age 18-70 years old, female and male	1. Pregnancy or breast-feeding women
2. Pathological confirmed adenocarcinoma	2. Pathological confirmed signet ring cell carcinoma
3. Clinical stage T1-3bN0, tumor maximum diameter less than 4cm	3. Clinical stage T1N0 and can be resected locally
4. The distance from anal verge less than 5 cm	4. History of other malignancies within 5 years
5. Without distance metastases	5. Known history of severe neurological or mental illness
6. KPS >=70	6. Serious medical illness, such as severe mental disorders, cardiac disease, uncontrolled infection, etc.
7. With good compliance	7. Immunodeficiency disease or long-term using of immunosuppressive agents
8. Microsatellite repair status is MSS/pMMR	8. Uncontrolled infection which needs systemic therapy
9. Without previous anti-cancer therapy or immunotherapy	9. Baseline blood and biochemical indicators do not meet the following criteria: neutrophils≥1.5×10^9/L, Hb≥90g/L, PLT≥100×10^9/L, ALT/AST ≤2.5 ULN, Cr≤ 1 ULN
10. Signed the inform consent	10. DPD deficiency
	11. Allergic to any component of the therapy

Treatment

Patients will first receive a total dose of 25Gy to the pelvic PTV delivered in fractions of 5 Gy. Almost two weeks after the last day of radiotherapy, four cycles of CAPOX chemotherapy and PD-1 antibody (Toripalimab) treatment will be performed. The CAPOX regimen consists of capecitabine and oxaliplatin, is repeated every three weeks. Oxaliplatin (130mg/m2) and Toripalimab (240 mg) will be administered intravenously on day 1. Capecitabine is administered orally at 1000 mg/m2 twice daily for 14 consecutive days. After the last cycle neoadjuvant treatment, response evaluation is possible to performed. IF the patients achieve cCR, a W&W strategy can be considered according to the decision of the patients. Meanwhile， patients with an incomplete response outcome will receive either local excision or total mesorectal excision (TME), determined by the multidisciplinary team.

Study endpoints

The primary endpoint is the CR rate, including rate of pCR and cCR. The CR rate is defined as the proportion of patients who achieve CR after preoperative therapy. Patients with the pCR status should have no viable tumour cells on the resected specimen. The cCR status is defined as undetectable tumour signs after neoadjuvant therapy through clinical examinations, including magnetic resonance imaging (MRI) of the pelvis, endoscopy, and digital rectal exam (DRE). The secondary endpoints include the organ preservation rate, 3-year local recurrence free survival (LRFS) rate, 3-year disease free survival (DFS) rate**,** 3-year overall survival (OS) rate, grade 3-4 adverse effects rate, and patients' quality of life.

For peer review only

Assessment

Regular examinations at the following time points will be performed: baseline, before the second PD-1 monoclonal antibody, after the last cycle of neoadjuvant treatment but prior to surgery and at every visit during the follow-up. Patients will undergo response evaluation with MRI of the pelvis, computerized tomography (CT) of chest and abdomen, DRE, blood routine examination, liver and renal function tests, myocardial enzymogram, thyroid hormone, adrenal hormone, and serum tumour markers.

Radiological assessment will be according to the Response Evaluation Criteria in Solid Tumours (RECIST 1.1)[23] and immune Response Evaluation Criteria in Solid Tumours (iRECIST)[24]. The pathological evaluation is mainly used to evaluate the degree of tumour regression on the gross resected specimen. Pathological tumour regression grade (pTRG) will be evaluated according to the 8th American Joint Committee on Cancer (AJCC) Staging Manual by two independent pathologists.

Adverse effects will be assessed according to the Common Terminology Criteria for Adverse Event (CTCAE) v5.0, patients' quality of life (QoL) will be evaluated using the EORTC QLQ-C30 and EORTC QLQ-CR29 scales. Additionally, the evaluation of anorectal and bowel function will be based on the Wexner score and LARS score[25, 26].

Sample size calculation

This study is a prospective, single-arm, phase II clinical study. The primary endpoint is the CR rate. According to the previous reports, the reference CR rate is about 45% (null hypothesis, p0), and we assumed that the CR rate in this study can be increased to 70% (alternative hypothesis, p1). With the statistical power of 0.8 (β=0.2), the type I error rate of 0.05 (α= 0.05), approximately 30 patients will be required in this study. Considering the dropout rate of 10%, the sample size in this study will be 34 cases.

Follow-up

The follow-up will be carried out every three months for at least three years postoperatively. The follow-up contents include the physical examination, tumour marker examination, and radiological examinations. MRI and CT scans will be performed alternately every three months. Colon endoscopy, late adverse effects collection, QoL assessment using EORTC questionnaires QLQ-C30 and QLQ-CR29, anorectal and bowel function using Wexner score and LARS score will be performed every 6 months. Colonoscopy, late adverse effects collection, QoL assessment (EORTC QLQ-C30/CR29), Wexner score and LARS score will be performed every 6 months. The events of local recurrence, distant metastasis and death will be recorded in detail.

Discussion

Direct surgery has traditionally been recommended as the standard treatment for early rectal cancer. However, the apparent benefits of nCRT in LARC patients have recently hastened interest in the application of nCRT for early rectal cancer patients. Initially, several retrospective studies[27, 28] revealed that the local tumour control of nCRT followed by TEM was comparable to that of direct TME surgery for T2–3 rectal cancer, and this finding was subsequently confirmed by a prospective

For peer review only

multi-institutional phase II ACOSOG Z6041 study[29]. In this study, 76 patients with T2N0 low-stage rectal cancer who had local resection after nCRT were enrolled. Among the included patients, the 3-year-DFS rate was 88.2%, the local recurrence rate was 5%, the organ-preservation rate was 91%, and 64% patients with cT2N0 tumours were downstaged to ypT0/T1 after nCRT, suggesting that nCRT might be considered as an organ-preserving alternative in low early rectal cancer patients, especially for those with difficulty in anus preservation.

In recent years, the neoadjuvant treatment paradigm for rectal cancer has gradually expanded from locally advanced stage to early-stage patients. At the 2022 ASCO Annual Meeting, four clinical studies exploring the efficiency of chemoradiotherapy with PD-1/PD-L1 inhibitors in early rectal cancer were intensively reported. The Spanish TAUTEM study compared the efficacy of preoperative CRT and TEM with conventional radical TME surgery in T2–T3s (superficial) N0, M0 (T2T3sN0M0) rectal cancer. Preliminary results showed that nCRT combined with TEM treatment obtained a high pCR rate (44.3%), with a high CRT compliance (98.8%) and low morbidity[30]. Meanwhile, the STAR-TREC phase II trial adopted patients with mrT1-3b N0 M0, $\leq$ 40 mm in tumour diameter, who were randomized to TME group, organ preservation followed SCRT (5 x 5 Gy) group or organ preservation followed LCRT (25 x 2 Gy + capecitabine) group. The results confirmed that nCRT was well tolerated, the 24-month local recurrence rate (LRT) was similar to the TME group, and organ preservation was achieved in 60% of patients[31]. The results of both TAUTEM and STAR-TREC studies have shown that CRT combined with local resection can achieve ideal organ preservation for early rectal cancer.

Organ preservation after reaching cCR on neoadjuvant therapy is gaining interest for early rectal cancer, and the following two studies are based on the W&W strategy after increasing the dose of radiotherapy to achieve CR. The OPERA trial from Europe showed that the "W&W" strategy can be adopted to preserve the organ for patients with cT2-T3 rectal cancer who reached a cCR after nCRT. And the organ preservation rates of contact X-ray brachytherapy (CXB) boost group and external beam radiation therapy (EBRT) boost group were 81% and 59%, respectively, suggesting that intracavitary radiotherapy may be a promising new paradigm for organ preservation in cT2-3 rectal cancer[32]. In addition, another ongoing phase II clinical trial (WW2 study) recruited 107 patients with resectable cT1-3N0-1M0 low rectal cancer[33]. Patients first received radiotherapy with 62Gy/28fx to the tumour and 50.4Gy/28fx to the regional lymph nodes. Meanwhile, capecitabine (825 mg/m^2, Bid) was administrated concurrently with radiotherapy. If patients achieved cCR after chemoradiation, the W&W strategy can be performed, surgery would be delivered for those with residual tumour or regrowth after cCR. The results showed that a total of 92 patients (86%) had cCR loco-regionally and were allocated to observation. The actuarial estimate of 2-year locoregional control from start of observation was 73.8% in patients with cCR, and the 30-month metastasis-free survival (MFS) and overall survival (OS) rates were 85.4% and 94.8%, respectively. The WW2 study confirmed the efficacy and safety of high-dose CRT, allowing patients with early rectal cancer to achieve radical results through chemoradiation and thus preserve the rectum.

However, the vast majority of neoadjuvant researches are based on traditional chemoradiotherapy, a relatively few studies using nCRT combined with PD-1/PD-L1 antibody in the neoadjuvant treatment of early rectal cancer. Nevertheless, one phase II study from Changhai Hospital was reported at the 2022 ASCO meeting[34]. In this study, 23 patients with T1-3aN0-1 low rectal cancer were included,

For peer review only

and received 2 cycles of Sintilimab concurrently with long-course radiotherapy, followed by 6 cycles of Sintilimab combined with capecitabine or CAPOX chemotherapy. 13 (56.5%) patients had an initial tumour stage of cT2, and 16(69.6%) were node positive. The results showed that of 23 patients, 10 (43.2%, 10/23) reached cCR, 2(20%, 2/10) reached pCR, and the CR (cCR+pCR) rate was 52.2% (12/23). In addition, the anus preservation rate was 95.5% (21/22), and the grade 3-4 toxicities were 17.4%. Thus, this study suggested that LCRT combined with PD-1 inhibitor could be one of the highly effective and less toxic treatment options for early and low rectal cancer when organ preservation is difficult with surgery.

Like the Changhai study mentioned above, most studies of radiotherapy combined with PD-1/PD-L1 inhibitors in rectal cancer are based on LCRT. Take VOLTAGE-A study[35] as an example, it performed 5 cycles of nivolumab after LCRT in LARC patients sand finally found that 11 of 37 (28%) MSS patients reached pCR, 3 (8%) reached near pCR, 1 reached cCR and adopted the W&W strategy. Additionally, there were another 3 of 5 (60%) microsatellite instability-high (MSI-H) patients reached pCR, and the adverse effects can be alleviated without affecting the surgical implementation. Compared with LCRT, SCRT combined with chemotherapy are also commonly used and can achieve similar outcomes for LARC patients. The Polish II 、 RAPIDO and STELLAR study have fully demonstrated the excellent efficacy of SCRT combined with chemotherapy[6, 36, 37]. From the perspective of radiotherapy combined with immunotherapy, SCRT may be more advantageous. It has been demonstrated that hypofractionated radiotherapy has less effect on peripheral blood lymphocytes in patients with locally advanced pancreatic cancer[38]. And hypofractionated radiotherapy can inhibit the recruitment of myeloid-derived suppressor cells (MDSC) to tumours, reduce PD-L1 expression on the tumour surface, and obtain better tumour growth inhibition rate than conventional fractionation in mice[39]. When combined with immunotherapy, abscopal effect can be observed[40]. These above findings provide a theoretical basis for the clinical trials of SCRT combined with PD-1/PD-L1 antibody and suggest the prospect of their application. So far, a number of clinical trials of LCRT combined with PD-1/PD-L1 inhibitors are currently ongoing, including NCT04109755, NCT03503630, and NCT04130854, while few studies on SCRT combined with immunotherapy for early rectal cancer have been reported yet. In our previous study, we have explored the model of SCRT combined with PD-1 inhibitor in LARC patients and showed high tumour regression efficacy with a pCR rate of 60.7%[20]. Meanwhile, we have also accumulated sufficient experience in the application and safety management of this regimen. Therefore, on the basis of others' and our own researches, we will carry out this study to explore the efficacy and safety of SCRT combined with chemotherapy and PD-1 inhibitor in low early rectal cancer patients.

In conclusion, the increasing demand for organ preservation and the excellent tumour regression has made neoadjuvant therapy an indispensable role in the treatment for low early-stage rectal cancer patients. In this context, TORCH-E is designed as a prospective, multi-center, single-arm, phase II trial of SCRT combined with CAPOX and Toripalimab in patients with early rectal cancer to investigate whether the addition of PD-1 inhibitor to nCRT can bring improved anal preservation, better prognosis, and good tolerance. We have reasons to believe the results of this study will help testify the efficacy and safety of the combination of PD-1 monotherapy and chemotherapy with SCRT, enhance the current understanding of clinical practice in the treatment of early low rectal cancer and pave the way for large scale clinical trial in the further.

For peer review only

Abbreviations

CAPOX: Capecitabine plus oxaliplatin; cCR: Clinical complete response; CR: Clinical response; CRC: Colorectal cancer; DRE: Digital rectal examination; DFS: Disease free survival; LARC: Locally advanced rectal cancer; LCRT: Long-course radiotherapy; LRFS: Local recurrence free survival; MDSC: Myeloid-derived suppressor cells; MRI: Magnetic resonance imaging; MSI-H: Microsatellite instability-high; pMMR : Proficient mismatch repair; MSS: Microsatellite- stable; NCCN: National Comprehensive Cancer Network; nCRT: Neoadjuvant chemoradiotherapy; OS: Overall survival; pCR: Pathological complete response; PD-1: programmed cell death-1; PD-L1: programmed cell death-ligand 1; QoL: Quality of life; RECIST: Response evaluation criteria in solid tumours; SCRT: Short-course radiotherapy; TME: Total mesorectal excision; TNT: Total neoadjuvant therapy; TRG: Tumour regression grade; W&W: Watch and Wait.

Declarations

Ethics approval and consent to participate

This study was conducted in compliance with Declaration of Helsinki principles. All procedures involving human subjects were approved by the Ethics Committee of Fudan University Shanghai Cancer Center (Approval Number: 2209261-18). All patients signed informed consent forms before recruitment.

Consent for publish

Not applicable.

Availability of data and materials

Not applicable. The data (such as efficacy and toxicity) produced during and after the trial are available from the corresponding author upon reasonable request.

Competing interests

The authors declare that they have no competing interests.

Funding

This study was supported by Shanghai Junshi Biomedical Technology Co., Ltd. (Project protocol No. JS001-ISS-C0402), the National Natural Science Foundation of China (Grant No. 82102978) and the National Key R&D Program of China (Grant No.2022YFC2503700, 2022YFC2503702).

Authors' contributions

Prof. FX, ZZ, and XL designed the clinical study. YC, YW1 (Yaqi Wang), MZ, RY, WY, SZ and FX made substantial contributions to the organization of this study. HZ, JW, LS, YW2 (Yan Wang), XL, SC, ZZ and FX were responsible for patient recruitment. YC and FX contributed to the manuscript drafting and revising. All authors gave final approval the version of the protocol, and it was also approved by local researchers of the participating centers.

Acknowledgements

For peer review only

We would like to thank Shanghai Junshi Biomedical Technology Co., Ltd. for their drug support and having purchased liability insurance for clinical trial subjects.

References

1. Heald RJ, Ryall RD: Recurrence and survival after total mesorectal excision for rectal cancer. Lancet 1986, 1(8496):1479-1482.
2. Wiltink LM, Marijnen CA, Meershoek-Klein Kranenbarg E, van de Velde CJ, Nout RA: A comprehensive longitudinal overview of health-related quality of life and symptoms after treatment for rectal cancer in the TME trial. Acta Oncol 2016, 55(4):502-508.
3. Cercek A, Roxburgh CSD, Strombom P, Smith JJ, Temple LKF, Nash GM, Guillem JG, Paty PB, Yaeger R, Stadler ZK et al: Adoption of Total Neoadjuvant Therapy for Locally Advanced Rectal Cancer. JAMA Oncol 2018, 4(6):e180071.
4. Garcia-Aguilar J, Chow OS, Smith DD, Marcet JE, Cataldo PA, Varma MG, Kumar AS, Oommen S, Coutsoftides T, Hunt SR et al: Effect of adding mFOLFOX6 after neoadjuvant chemoradiation in locally advanced rectal cancer: a multicentre, phase 2 trial. Lancet Oncol 2015, 16(8):957-966.
5. van der Valk MJM, Hilling DE, Bastiaannet E, Meershoek-Klein Kranenbarg E, Beets GL, Figueiredo NL, Habr-Gama A, Perez RO, Renehan AG, van de Velde CJH et al: Long-term outcomes of clinical complete responders after neoadjuvant treatment for rectal cancer in the International Watch & Wait Database (IWWD): an international multicentre registry study. Lancet 2018, 391(10139):2537-2545.
6. Bahadoer RR, Dijkstra EA, van Etten B, Marijnen CAM, Putter H, Kranenbarg EM, Roodvoets AGH, Nagtegaal ID, Beets-Tan RGH, Blomqvist LK et al: Short-course radiotherapy followed by chemotherapy before total mesorectal excision (TME) versus preoperative chemoradiotherapy, TME, and optional adjuvant chemotherapy in locally advanced rectal cancer (RAPIDO): a randomised, open-label, phase 3 trial. Lancet Oncol 2021, 22(1):29-42.
7. Conroy T, Bosset JF, Etienne PL, Rio E, Francois E, Mesgouez-Nebout N, Vendrely V, Artignan X, Bouche O, Gargot D et al: Neoadjuvant chemotherapy with FOLFIRINOX and preoperative chemoradiotherapy for patients with locally advanced rectal cancer (UNICANCER-PRODIGE 23): a multicentre, randomised, open-label, phase 3 trial. Lancet Oncol 2021, 22(5):702-715.
8. Robert C, Ribas A, Schachter J, Arance A, Grob JJ, Mortier L, Daud A, Carlino MS, McNeil CM, Lotem M et al: Pembrolizumab versus ipilimumab in advanced melanoma (KEYNOTE-006): post-hoc 5-year results from an open-label, multicentre, randomised, controlled, phase 3 study. Lancet Oncol 2019, 20(9):1239-1251.
9. Leighl NB, Hellmann MD, Hui R, Carcereny E, Felip E, Ahn MJ, Eder JP, Balmanoukian AS, Aggarwal C, Horn L et al: Pembrolizumab in patients with advanced non-small-cell lung cancer (KEYNOTE-001): 3-year results from an open-label, phase 1 study. Lancet Respir Med 2019, 7(4):347-357.
10. Andre T, Shiu KK, Kim TW, Jensen BV, Jensen LH, Punt C, Smith D, Garcia-Carbonero R, Benavides M, Gibbs P et al: Pembrolizumab in Microsatellite-Instability-High Advanced Colorectal Cancer. N Engl J Med 2020, 383(23):2207-2218.
11. André T, Shiu KK, Kim TW, Jensen BV, Jensen LH, Punt C, Smith D, Garcia-Carbonero R,

For peer review only

Benavides M, Gibbs P et al: Pembrolizumab in Microsatellite-Instability-High Advanced Colorectal Cancer. N Engl J Med 2020, 383(23):2207-2218.

12. Overman MJ, Lonardi S, Wong KYM, Lenz HJ, Gelsomino F, Aglietta M, Morse MA, Van Cutsem E, McDermott R, Hill A et al: Durable Clinical Benefit With Nivolumab Plus Ipilimumab in DNA Mismatch Repair-Deficient/Microsatellite Instability-High Metastatic Colorectal Cancer. J Clin Oncol 2018, 36(8):773-779.
13. Dovedi SJ, Illidge TM: The antitumor immune response generated by fractionated radiation therapy may be limited by tumor cell adaptive resistance and can be circumvented by PD-L1 blockade. Oncoimmunology 2015, 4(7):e1016709.
14. Dovedi SJ, Adlard AL, Lipowska-Bhalla G, McKenna C, Jones S, Cheadle EJ, Stratford IJ, Poon E, Morrow M, Stewart R et al: Acquired resistance to fractionated radiotherapy can be overcome by concurrent PD-L1 blockade. Cancer research 2014, 74(19):5458-5468.
15. Bando H, Tsukada Y, Inamori K, Togashi Y, Koyama S, Kotani D, Fukuoka S, Yuki S, Komatsu Y, Homma S et al: Preoperative Chemoradiotherapy plus Nivolumab before Surgery in Patients with Microsatellite Stable and Microsatellite Instability-High Locally Advanced Rectal Cancer. Clin Cancer Res 2022, 28(6):1136-1146.
16. Tamberi S, Grassi E, Zingaretti C, G P: Phase ii study of capecitabine plus concomitant radiation therapy followed by durvalumab (Medi4736) as preoperative treatment in rectal cancer: Pandora study final results. ASCO, 2022. Chicago.
17. George TJ, Yothers G, Jacobs SA, Finley GG, Wade JL, CMSPR L: Phase ii study of durvalumab following neoadjuvant chemort in operable rectal cancer: Nsabp fr-2. ASCO GI, 2022. San Francisco.
18. Wang Y, Shen L, Wan J, Zhang H, Wu R, Wang J, Wang Y, Xu Y, Cai S, Zhang Z et al: Short-course radiotherapy combined with CAPOX and Toripalimab for the total neoadjuvant therapy of locally advanced rectal cancer: a randomized, prospective, multicentre, double-arm, phase II trial (TORCH). BMC Cancer 2022, 22(1):274.
19. Wang Y, Xia F, Shen L, Wan J, Zhang H, Wu R, Wang J, Wang Y, Xu Y, Cai S et al: Short-course radiotherapy combined with capox and toripalimab for the total neoadjuvant therapy of locally advanced rectal cancer: Preliminary findings from a randomized, prospective, multicenter, double-arm, phase ii trial (Torch). ASCO, 2022. Chicago.
20. Wang Y, Shen L, Wan J, Zhang H, Wu R, Wang J, Wang Y, Xu Y, Cai S, Zhang Z et al: Neoadjuvant chemoradiotherapy combined with immunotherapy for locally advanced rectal cancer: A new era for anal preservation. Front Immunol 2022, 13:1067036.
21. Shamseddine A, Zeidan YH, El Husseini Z, Kreidieh M, Al Darazi M, Turfa R, Kattan J, Khalifeh I, Mukherji D, Temraz S et al: Efficacy and safety-in analysis of short-course radiation followed by mFOLFOX-6 plus avelumab for locally advanced rectal adenocarcinoma. Radiat Oncol 2020, 15(1):233.
22. Sheng Dai, Fei Wang, Yanbin Shen, Liming Shi, Lingna Xu, Chuanxi Lai, Feixiang Zhang, Kangke Zhang, Dengyong Xu, Lina Shan et al: Efficacy and safety of neoadjuvant preoperative short-course radiation followed by envafolimab plus CAPEOX in microsatellite stable (MSS)/mismatch repair proficient (pMMR) locally advanced rectal cancer. ASCO GI, 2023. San Francisco.
23. Eisenhauer EA, Therasse P, Bogaerts J, Schwartz LH, Sargent D, Ford R, Dancey J, Arbuck S, Gwyther S, Mooney M et al: New response evaluation criteria in solid tumours: revised

For peer review only

RECIST guideline (version 1.1). Eur J Cancer 2009, 45(2):228-247.

24. Seymour L, Bogaerts J, Perrone A, Ford R, Schwartz LH, Mandrekar S, Lin NU, Litiere S, Dancey J, Chen A et al: iRECIST: guidelines for response criteria for use in trials testing immunotherapeutics. Lancet Oncol 2017, 18(3):e143-e152.

25. Jorge JM, Wexner SD: Etiology and management of fecal incontinence. Dis Colon Rectum 1993, 36(1):77-97.

26. Emmertsen KJ, Laurberg S: Low anterior resection syndrome score: development and validation of a symptom-based scoring system for bowel dysfunction after low anterior resection for rectal cancer. Ann Surg 2012, 255(5):922-928.

27. Borschitz T, Wachtlin D, Mohler M, Schmidberger H, Junginger T: Neoadjuvant chemoradiation and local excision for T2-3 rectal cancer. Ann Surg Oncol 2008, 15(3):712-720.

28. Nair RM, Siegel EM, Chen DT, Fulp WJ, Yeatman TJ, Malafa MP, Marcet J, Shibata D: Long-term results of transanal excision after neoadjuvant chemoradiation for T2 and T3 adenocarcinomas of the rectum. J Gastrointest Surg 2008, 12(10):1797-1805; discussion 1805-1796.

29. Garcia-Aguilar J, Renfro LA, Chow OS, Shi Q, Carrero XW, Lynn PB, Thomas CR, Jr., Chan E, Cataldo PA, Marcet JE et al: Organ preservation for clinical T2N0 distal rectal cancer using neoadjuvant chemoradiotherapy and local excision (ACOSOG Z6041): results of an open-label, single-arm, multi-institutional, phase 2 trial. Lancet Oncol 2015, 16(15):1537-1546.

30. Serra-Aracil X, Pericay C, Badia-Closa J, Golda T, Biondo S, Hernandez P, Targarona E, Borda-Arrizabalaga N, Reina A, Delgado S et al: Short-term outcomes of chemoradiotherapy and local excision versus total mesorectal excision in T2-T3ab,N0,M0 rectal cancer: a multicentre randomised, controlled, phase III trial (the TAU-TEM study). Ann Oncol 2023, 34(1):78-90.

31. Simon Parkinson Bach, Johannes de Wilt, Femke Peters, Karen-Lise Garm Spindler, Ane Appelt, Mark Teo, Victoria Homer, Natalie Abbott, lan Geh, Stephan Korsgen et al: Can we save the rectum by watchful waiting or transanal surgery following (chemo)Radiotherapy versus total mesorectal excision for early rectal cancer? ASCO, 2022. Chicago.

32. Gerard JP, Barbet N, Schiappa R, Magne N, Martel I, Mineur L, Deberne M, Zilli T, Dhadda A, Myint AS et al: Neoadjuvant chemoradiotherapy with radiation dose escalation with contact x-ray brachytherapy boost or external beam radiotherapy boost for organ preservation in early cT2-cT3 rectal adenocarcinoma (OPERA): a phase 3, randomised controlled trial. Lancet Gastroenterol Hepatol 2023.

33. Jensen LH: Curative chemoradiation for low rectal cancer: primary clinical outcomes from a multicenter phase II trial. ASCO, 2022. Chicago.

34. Zhou L, Yu G, Y S: The clinical efficacy and safety of neoadjuvant chemoradiation therapy with immunotherapy for the organ preservation of ultra low rectal cancer: A single arm and open label exploratory study. ASCO, 2022. Chicago.

35. Yuki S, Bando H, Y T: Short-term results of VOLTAGE-A: Nivolumab monotherapy and subsequent radical surgery following pre- operative chemoradiotherapy in patients with microsatellite stable and microsatellite instability-high locally advanced rectal cancer. ASCO, 2020. Chicago.

36. Jin J, Tang Y, Hu C, Jiang LM, Jiang J, Li N, Liu WY, Chen SL, Li S, Lu NN et al:

For peer review only

Multicenter, Randomized, Phase III Trial of Short-Term Radiotherapy Plus Chemotherapy Versus Long-Term Chemoradiotherapy in Locally Advanced Rectal Cancer (STELLAR). J Clin Oncol 2022, 40(15):1681-1692.

37. Bujko K, Wyrwicz L, Rutkowski A, Malinowska M, Pietrzak L, Krynski J, Michalski W, Oledzki J, Kusnierz J, Zajac L et al: Long-course oxaliplatin-based preoperative chemoradiation versus 5 x 5 Gy and consolidation chemotherapy for cT4 or fixed cT3 rectal cancer: results of a randomized phase III study. Ann Oncol 2016, 27(5):834-842.

38. Crocenzi T, Cottam B, Newell P, Wolf RF, Hansen PD, Hammill C, Solhjem MC, To YY, Greathouse A, Tormoen G et al: A hypofractionated radiation regimen avoids the lymphopenia associated with neoadjuvant chemoradiation therapy of borderline resectable and locally advanced pancreatic adenocarcinoma. J Immunother Cancer 2016, 4:45.

39. Lan J, Li R, Yin LM, Deng L, Gui J, Chen BQ, Zhou L, Meng MB, Huang QR, Mo XM et al: Targeting Myeloid-derived Suppressor Cells and Programmed Death Ligand 1 Confers Therapeutic Advantage of Ablative Hypofractionated Radiation Therapy Compared With Conventional Fractionated Radiation Therapy. Int J Radiat Oncol Biol Phys 2018, 101(1):74-87.

40. Dewan MZ, Galloway AE, Kawashima N, Dewyngaert JK, Babb JS, Formenti SC, Demaria S: Fractionated but not single-dose radiotherapy induces an immune-mediated abscopal effect when combined with anti-CTLA-4 antibody. Clin Cancer Res 2009, 15(17):5379-5388.

Figure Legends

Figure.1 Flow chart of the TORCH-E study. Note: SCRT, short- course radiotherapy; mAb, monoclonal antibody; CAPOX, capecitabine plus oxaliplatin; cCR: clinical complete response; CR: clinical response; TME: total mesorectal excision.

For peer review only

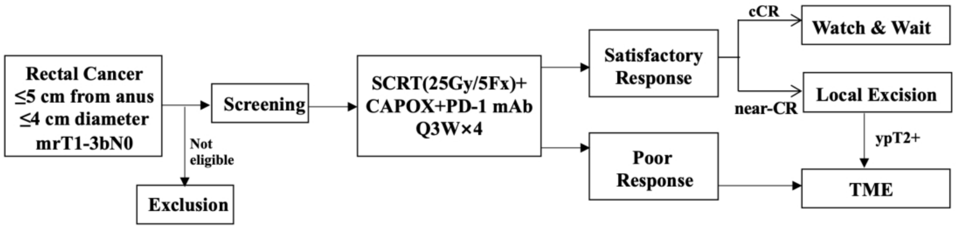

Flow chart of the TORCH-E study.

338x190mm (150 x 150 DPI)

STANDARD PROTOCOL ITEMS: RECOMMENDATIONS FOR INTERVENTIONAL TRIALS

SPIRIT 2013 Checklist: Recommended items to address in a clinical trial protocol and related documents*

Section/item	Item No	Description	Addressed on page number
**Administrative information**			
Title	1	Descriptive title identifying the study design, population, interventions, and, if applicable, trial acronym	Page1, paragraph1
Trial registration	2a	Trial identifier and registry name. If not yet registered, name of intended registry	Page2, paragraph4
	2b	All items from the World Health Organization Trial Registration Data Set	NA
Protocol version	3	Date and version identifier	Ver 1.0 (2023.05.16)
Funding	4	Sources and types of financial, material, and other support	Page9, paragraph8
Roles and responsibilities	5a	Names, affiliations, and roles of protocol contributors	Page9, paragraph7
	5b	Name and contact information for the trial sponsor	Page1, paragraph4
	5c	Role of study sponsor and funders, if any, in study design; collection, management, analysis, and interpretation of data; writing of the report; and the decision to submit the report for publication, including whether they will have ultimate authority over any of these activities	Page9, paragraph7
	5d	Composition, roles, and responsibilities of the coordinating centre, steering committee, endpoint adjudication committee, data management team, and other individuals or groups overseeing the trial, if applicable (see Item 21a for data monitoring committee)	Available on request

For peer review only - http://bmjopen.bmj.com/site/about/guidelines.xhtml

**Introduction**			
Background and rationale	6a	Description of research question and justification for undertaking the trial, including summary of relevant studies (published and unpublished) examining benefits and harms for each intervention	Page3-4
	6b	Explanation for choice of comparators	Page4, paragraph1
Objectives	7	Specific objectives or hypotheses	Page4, paragraph2
Trial design	8	Description of trial design including type of trial (eg, parallel group, crossover, factorial, single group), allocation ratio, and framework (eg, superiority, equivalence, noninferiority, exploratory)	Page4, paragraph3
**Methods: Participants, interventions, and outcomes**			
Study setting	9	Description of study settings (eg, community clinic, academic hospital) and list of countries where data will be collected. Reference to where list of study sites can be obtained	Page4, paragraph4
Eligibility criteria	10	Inclusion and exclusion criteria for participants. If applicable, eligibility criteria for study centres and individuals who will perform the interventions (eg, surgeons, psychotherapists)	Page5, Table1
Interventions	11a	Interventions for each group with sufficient detail to allow replication, including how and when they will be administered	Page5, paragraph3
	11b	Criteria for discontinuing or modifying allocated interventions for a given trial participant (eg, drug dose change in response to harms, participant request, or improving/worsening disease)	Page5-6
	11c	Strategies to improve adherence to intervention protocols, and any procedures for monitoring adherence (eg, drug tablet return, laboratory tests)	Page5-6
	11d	Relevant concomitant care and interventions that are permitted or prohibited during the trial	Page5-6
Outcomes	12	Primary, secondary, and other outcomes, including the specific measurement variable (eg, systolic blood pressure), analysis metric (eg, change from baseline, final value, time to event), method of aggregation (eg, median, proportion), and time point for each outcome. Explanation of the clinical relevance of chosen efficacy and harm outcomes is strongly recommended	Page5, paragraph4
Participant timeline	13	Time schedule of enrolment, interventions (including any run-ins and washouts), assessments, and visits for participants. A schematic diagram is highly recommended (see Figure)	Figure1

For peer review only - http://bmjopen.bmj.com/site/about/guidelines.xhtml

Sample size	14	Estimated number of participants needed to achieve study objectives and how it was determined, including clinical and statistical assumptions supporting any sample size calculations	Page6, paragraph4
4Recruitment	15	Strategies for achieving adequate participant enrolment to reach target sample size	Page6, paragraph4

Methods: Assignment of interventions (for controlled trials)

Allocation:

Sequence generation	16a	Method of generating the allocation sequence (eg, computer-generated random numbers), and list of any factors for stratification. To reduce predictability of a random sequence, details of any planned restriction (eg, blocking) should be provided in a separate document that is unavailable to those who enrol participants or assign interventions	Available on request
Allocation concealment mechanism	16b	Mechanism of implementing the allocation sequence (eg, central telephone; sequentially numbered, opaque, sealed envelopes), describing any steps to conceal the sequence until interventions are assigned	Available on request
Implementation	16c	Who will generate the allocation sequence, who will enrol participants, and who will assign participants to interventions	Prof. Zhen Zhang
Blinding (masking)	17a	Who will be blinded after assignment to interventions (eg, trial participants, care providers, outcome assessors, data analysts), and how	NA
	17b	If blinded, circumstances under which unblinding is permissible, and procedure for revealing a participant's allocated intervention during the trial	NA

Methods: Data collection, management, and analysis

Data collection methods	18a	Plans for assessment and collection of outcome, baseline, and other trial data, including any related processes to promote data quality (eg, duplicate measurements, training of assessors) and a description of study instruments (eg, questionnaires, laboratory tests) along with their reliability and validity, if known. Reference to where data collection forms can be found, if not in the protocol	Page6, paragraph1-3
	18b	Plans to promote participant retention and complete follow-up, including list of any outcome data to be collected for participants who discontinue or deviate from intervention protocols	Page6, paragraph5

Data management	19	Plans for data entry, coding, security, and storage, including any related processes to promote data quality (eg, double data entry; range checks for data values). Reference to where details of data management procedures can be found, if not in the protocol	Available on request
Statistical methods	20a	Statistical methods for analysing primary and secondary outcomes. Reference to where other details of the statistical analysis plan can be found, if not in the protocol	Available on request
	20b	Methods for any additional analyses (eg, subgroup and adjusted analyses)	Available on request
	20c	Definition of analysis population relating to protocol non-adherence (eg, as randomised analysis), and any statistical methods to handle missing data (eg, multiple imputation)	Available on request
**Methods: Monitoring**			
Data monitoring	21a	Composition of data monitoring committee (DMC); summary of its role and reporting structure; statement of whether it is independent from the sponsor and competing interests; and reference to where further details about its charter can be found, if not in the protocol. Alternatively, an explanation of why a DMC is not needed	Available on request
	21b	Description of any interim analyses and stopping guidelines, including who will have access to these interim results and make the final decision to terminate the trial	Available on request
Harms	22	Plans for collecting, assessing, reporting, and managing solicited and spontaneously reported adverse events and other unintended effects of trial interventions or trial conduct	NA
Auditing	23	Frequency and procedures for auditing trial conduct, if any, and whether the process will be independent from investigators and the sponsor	NA
**Ethics and dissemination**			
Research ethics approval	24	Plans for seeking research ethics committee/institutional review board (REC/IRB) approval	Page9, paragraph2
Protocol amendments	25	Plans for communicating important protocol modifications (eg, changes to eligibility criteria, outcomes, analyses) to relevant parties (eg, investigators, REC/IRBs, trial participants, trial registries, journals, regulators)	Available on request

Consent or assent	26a	Who will obtain informed consent or assent from potential trial participants or authorised surrogates, and how (see Item 32)	Dr. Yajie Chen
	26b	Additional consent provisions for collection and use of participant data and biological specimens in ancillary studies, if applicable	Available on request
Confidentiality	27	How personal information about potential and enrolled participants will be collected, shared, and maintained in order to protect confidentiality before, during, and after the trial	Available on request
Declaration of interests	28	Financial and other competing interests for principal investigators for the overall trial and each study site	Page9, paragraph5
Access to data	29	Statement of who will have access to the final trial dataset, and disclosure of contractual agreements that limit such access for investigators	Page9, paragraph4
Ancillary and post-trial care	30	Provisions, if any, for ancillary and post-trial care, and for compensation to those who suffer harm from trial participation	Page4, paragraph4
Dissemination policy	31a	Plans for investigators and sponsor to communicate trial results to participants, healthcare professionals, the public, and other relevant groups (eg, via publication, reporting in results databases, or other data sharing arrangements), including any publication restrictions	Medical conference
	31b	Authorship eligibility guidelines and any intended use of professional writers	Available on request
	31c	Plans, if any, for granting public access to the full protocol, participant-level dataset, and statistical code	Available on request
**Appendices**			
Informed consent materials	32	Model consent form and other related documentation given to participants and authorised surrogates	Available on request
Biological specimens	33	Plans for collection, laboratory evaluation, and storage of biological specimens for genetic or molecular analysis in the current trial and for future use in ancillary studies, if applicable	NA

*It is strongly recommended that this checklist be read in conjunction with the SPIRIT 2013 Explanation & Elaboration for important clarification on the items. Amendments to the protocol should be tracked and dated. The SPIRIT checklist is copyrighted by the SPIRIT Group under the Creative Commons "Attribution-NonCommercial-NoDerivs 3.0 Unported" license.

For peer review only - http://bmjopen.bmj.com/site/about/guidelines.xhtml

BMJ Open

Short-course radiotherapy combined with chemotherapy and PD-1 inhibitor in low-lying early rectal cancer: study protocol for a single-arm, multi-center, prospective, phase II trial (TORCH-E)

Journal:	*BMJ Open*
Manuscript ID	bmjopen-2023-076048.R1
Article Type:	Protocol
Date Submitted by the Author:	16-Aug-2023

Complete List of Authors:	Chen, Yajie; Fudan University Shanghai Cancer Center, Department of Radiation Oncology; Shanghai Medical College of Fudan University, Department of Oncology<br>Wang, Yaqi; Fudan University Shanghai Cancer Center, Department of Radiation Oncology; Shanghai Medical College of Fudan University, Department of Oncology<br>Zhang, Hui; Fudan University Shanghai Cancer Center, Department of Radiation Oncology; Shanghai Medical College of Fudan University, Department of Oncology<br>Wan, Juefeng; Fudan University Shanghai Cancer Center, Department of Radiation Oncology; Shanghai Medical College of Fudan University, Department of Oncology<br>Shen, Lijun; Fudan University Shanghai Cancer Center, Department of Radiation Oncology; Shanghai Medical College of Fudan University, Department of Oncology<br>Wang, Yan; Fudan University Shanghai Cancer Center, Department of Radiation Oncology; Shanghai Medical College of Fudan University, Department of Oncology<br>Zhou, Menglong; Fudan University Shanghai Cancer Center, Department of Radiation Oncology; Shanghai Medical College of Fudan University, Department of Oncology<br>Wu, Ruiyan; Fudan University Shanghai Cancer Center, Department of Radiation Oncology; Shanghai Medical College of Fudan University, Department of Oncology<br>Yang, Wang; Fudan University Shanghai Cancer Center, Department of Radiation Oncology<br>Zhou, Shujuan; Fudan University Shanghai Cancer Center, Department of Radiation Oncology<br>Cai, Sanjun; Shanghai Medical College of Fudan University, Department of Oncology; Fudan University Shanghai Cancer Center, Department of Colorectal Surgery<br>Li, Xinxiang; Shanghai Medical College of Fudan University, Department of Oncology; Fudan University Shanghai Cancer Center, Department of Colorectal Surgery<br>Zhang, Zhen; Fudan University Shanghai Cancer Center, Department of Radiation Oncology; Shanghai Medical College of Fudan University, Department of Oncology<br>Xia, Fan; Fudan University Shanghai Cancer Center, Department of

	Radiation Oncology; Shanghai Medical College of Fudan University, Department of Oncology
**Primary Subject Heading**:	Oncology
Secondary Subject Heading:	Gastroenterology and hepatology
Keywords:	GASTROENTEROLOGY, RADIOTHERAPY, ONCOLOGY

SCHOLARONE™
Manuscripts

Short-course radiotherapy combined with chemotherapy and PD-1 inhibitor in low-lying early rectal cancer: study protocol for a single-arm, multi-center, prospective, phase II trial (TORCH-E)

Yajie Chen[1,2], Yaqi Wang[1,2], Hui Zhang[1,2], Juefeng Wan[1,2], Lijun Shen[1,2], Yan Wang[1,2], Menglong Zhou[1,2], Ruiyan Wu[1,2], Wang Yang[1,2], Shujuan Zhou[1,2], Sanjun Cai[2,3], Xinxiang Li[2,3*], Zhen Zhang[1,2*] and Fan Xia[1,2*]

First author: Yajie Chen

***Corresponding author:**

Xinxiang Li, Department of Colorectal Surgery, Fudan University Shanghai Cancer Center, Shanghai, China. Email: xinxiangli@fudan.edu.cn

Zhen Zhang, Department of Radiation Oncology, Fudan University Shanghai Cancer Center, Shanghai, China. Email: zhen_zhang@fudan.edu.cn

Fan Xia, Department of Radiation Oncology, Fudan University Shanghai Cancer Center, Shanghai, China. Email: tcxiafan@hotmail.com

For peer review only

Abstract

Introduction: Current standard treatment for patients with early rectal cancer is radical surgical resection. Although radical surgery provides effective local tumor control, it also increases the mortality risk and considerable adverse effects, including bowel, bladder, sexual dysfunction and loss of anal function, especially in patients with low-lying rectal cancer. Recent studies have shown promising synergistic effects of the combination of immunotherapy (PD-1/PD-L1 inhibitors) and radiotherapy in improving tumor regression. For patients who reach a clinical complete response (cCR) after neoadjuvant therapy, a "Watch & Wait" (W&W) approach can be adopted to preserve anorectal function and improve quality of life. Thus, this study aims to explore the efficacy and safety of radiotherapy combined with chemotherapy and PD-1 antibody in low early rectal cancer patients.

Methods and analysis: TORCH-E study is designed as a multi-center, prospective, phase II trial of short-course radiotherapy (SCRT) combined with chemotherapy and PD-1 inhibitor in cT1-3bN0M0 low rectal cancer patients. The trial was initiated in December 2022 and is currently recruiting patients, with an anticipated completion of participant enrollment by June of the following year. The enrolled 34 patients will receive SCRT (25 Gy/5Fx), followed by four cycles of CAPOX chemotherapy and PD-1 antibody (Toripalimab), and finally receive surgery or the W&W strategy. The primary endpoint is the complete response (pCR plus cCR) rate. The secondary endpoints include organ preservation rate, 3-year local recurrence free survival (LRFS) rate, 3-year disease free survival (DFS) rate, 3-year overall survival (OS) rate, grade 3-4 adverse effects rate, and patients' quality of life (QoL).

Ethics and dissemination: This trial has been approved by the Ethics Committee of Fudan University Shanghai Cancer Center. Trial results will be disseminated via peer reviewed journals and conference presentations.

Trial registration number: NCT05555888 (ClinicalTrials.gov); Date of registration: September 22, 2022; URL of trial registry record: https://clinicaltrials.gov/ct2/show/NCT05555888

Strengths and limitations of this study:

1. This clinical trial is the first prospective, multi-center, phase II study using short-course radiotherapy combined with PD-1 inhibitor and chemotherapy in early low-lying rectal cancer.
2. This study aims to investigate the impact of short-course radiotherapy on tumor regression and its synergy with immunotherapy, differing from previous studies employing long-course radiotherapy.
3. The participants with early low rectal cancer can attain non-operative management (NOM) or minimally invasive surgery via a combination of radiotherapy, chemotherapy, and PD-1 inhibitor, aiming for optimal organ function preservation.
4. Limitation: this is a phase II clinical study involving a small number of patients, a phase III trial with a larger sample size will be necessary to validate the effectiveness and safety.

For peer review only

Introduction

The standard treatment for cT1-3bN0M0 (stage I-II) rectal cancer remains radical surgical resection. Despite effective local tumor control, surgical resection can elevate mortality risk and lead to notable adverse effects, such as high stoma rates, fecal incontinence, and urinary and sexual dysfunction[1], particularly in cases of low-lying rectal cancers. Most survivors encounter functional sequelae due to removal of the rectum, which significantly impairs their quality of life (QoL).

Neoadjuvant chemoradiotherapy (nCRT) is recognized for its potential to promote tumor regression, reduce local recurrence rates, and enhance sphincter preservation in locally advanced rectal cancer (LARC). Patients who reach a complete clinical response after nCRT can adopt the "Watch & Wait" (W&W) strategy to preserve the rectum[2], and the total neoadjuvant therapy (TNT) approach is also expected to decrease distant metastasis and improve long-term survival[3, 4]. The evident benefits of nCRT in LARC have spurred interest in its application for early rectal cancer. Initial retrospective studies indicated comparable local tumor control between nCRT followed by TEM and direct TME surgery for T2–3 rectal cancer[5, 6]. A subsequent prospective multi-institutional phase II ACOSOG Z6041 study validated this finding, showing favorable 3-year disease-free survival, organ preservation rates, and substantial tumor downstaging in patients with T2N0 low-stage rectal cancer who underwent local resection following nCRT[7]. These results suggest the potential of nCRT as an organ-preserving alternative for low early rectal cancer patients, particularly those encountering challenges in anus preservation.

In recent years, numerous clinical studies have extensively explored the nCRT treatment approach for early rectal cancer. The Spanish TAUTEM study compared preoperative CRT and TEM with conventional TME surgery in T2-T3s (superficial) N0, M0 rectal cancer, and found that combining nCRT with TEM achieved a high pCR rate (44.3%)[8]. Similarly, the STAR-TREC phase II trial adopted patients with mrT1-3b N0 M0, ≤ 40 mm tumor diameter. Randomized into TME, organ preservation followed by short-course radiotherapy (SCRT), or organ preservation followed by long-course chemoradiotherapy (LCRT) groups, the trial showed that nCRT was well-tolerated. The 24-month local recurrence rate (LRT) was comparable to the TME group, with 60% achieving organ preservation[9]. The TAUTEM and STAR-TREC studies collectively demonstrate the viability of CRT combined with local resection to achieve optimal organ preservation in early rectal cancer. Additionally, the OPERA trial and WW2 study validate high-dose CRT's efficacy and safety, enabling early rectal cancer patients to attain radical outcomes via chemoradiation, thereby preserving the rectum[10, 11].

Preclinical evidence suggests that combining radiotherapy with PD-1/PD-L1 antibodies can reshape the tumor microenvironment, counter immunosuppression, boost T-cell-derived antitumor cytokine secretion, and enhance radiotherapy's effectiveness[12, 13]. However, while many neoadjuvant studies focus on traditional chemoradiotherapy, only a few investigate the combination of nCRT with PD-1/PD-L1 antibodies for early rectal cancer. Notably, a Changhai Hospital phase II study presented at the 2022 ASCO meeting enrolled 23 patients with T1-3aN0-1 low rectal cancer[14]. They received concurrent Sintilimab and LCRT, followed by Sintilimab and chemotherapy. Among the patients, 43.2% achieved cCR, 20% achieved pCR, resulting in a combined CR rate of 52.2%. Anus preservation was at 95.5%, and grade 3-4 toxicities were 17.4%. These findings suggest that combining LCRT with PD-

1 inhibitors could offer a highly effective and less toxic treatment option for challenging cases of early and low rectal cancer where surgical preservation is difficult.

Similar to Changhai study, many investigations into radiotherapy combined with PD-1/PD-L1 inhibitors for rectal cancer focus on LCRT. Comparably, SCRT combined with chemotherapy has also demonstrated favorable outcomes for LARC patients in the Polish II, RAPIDO, and STELLAR studies [3, 15, 16]. From the perspective of radiotherapy combined with immunotherapy, SCRT could be advantageous. Hypofractionated radiotherapy has shown less impact on peripheral blood lymphocytes in locally advanced pancreatic cancer patients[17]. It can also curb myeloid-derived suppressor cell (MDSC) recruitment to tumors, decrease PD-L1 expression on tumor surfaces, and yield improved tumor growth inhibition rates compared to conventional fractionation in mice[18], even displaying abscopal effects when paired with immunotherapy[19]. These above findings provide a theoretical basis for the clinical trials of SCRT combined with PD-1/PD-L1 antibody and suggest the prospect of their application.

While there's a scarcity of reports on SCRT combined with immunotherapy for early rectal cancer, the TORCH study conducted by Fudan University adopted the TNT model showcased promising tumor regression efficacy with SCRT and PD-1 inhibitor in LARC patients[20, 21]. The results remarkably showed that 60.4% patients achieved CR, 60.7% achieved pCR， 78.6% reached major pathologic response (MPR), and the organ preservation rate was 88.9%[22]. Furthermore, two additional SCRT-based trials also demonstrated substantial pCR rates LARC patients[23, 24]. These prospective studies collectively suggest that integrating CRT, chemotherapy, and PD-1/PD-L1 antibodies for LARC patients could potentially yield higher tumor regression and CR rates compared to traditional chemoradiotherapy. Therefore, this approach holds promise for achieving favorable tumor responses and organ preservation rates when applied to low-lying early rectal cancer.

Based upon the above findings and our previous TORCH study, the treatment approach of combining PD-1 monotherapy with SCRT and chemotherapy is expected to remarkably improve tumor regression and CR rate, and provide more options for patients with early rectal cancer to achieve organ preservation. Moreover, about 95% of patients with rectal cancer have the proficient mismatch repair (pMMR)/ microsatellite-stable (MSS) type, which is not sensitive to PD-1 inhibitor alone. Therefore, we are conducting a phase II trial of the combination of short-course radiotherapy, CAPOX and Toripalimab for neoadjuvant therapy in pMMR/MSS, early and low rectal cancer, with the expectation to explore the efficacy and safety of this combination therapy.

Methods and analysis

Patient and public involvement

Neither patients nor the public will be involved in the design, recruitment, assessment and conduct of this study. The results will be disseminated via peer-reviewed scientific journals and conference presentations rather than specifically notified to a single patient.

Protocol version

Protocol V.2.0, modified on 14 August 2023.

For peer review only

Study design

The TORCH-E study is a multi-center, prospective phase II trial of short-term radiotherapy combined with chemotherapy and PD-1 inhibitor in early low rectal cancer. The enrolled patients will receive short-term radiotherapy (25 Gy/5Fx), followed by 4 cycles of CAPOX chemotherapy and PD-1 antibody (Toripalimab), and finally receive TME surgery or local excision (TEM). The complete response (pCR plus cCR) rate, organ preservation rate, long-term prognosis, and adverse effects will be analyzed. The scheme of study progress was showed in Fig. 1.

Trial organization

This trial is investigator initiated by the Department of Radiation Oncology, Fudan University Shanghai Cancer Center (FUSCC). Five other cancer centers in China are involved in the study, including Renji Hospital, Shanghai Changzheng Hospital, Fujian Cancer Hospital, the Second Affiliated Hospital Zhejiang University School of Medicine and the Second Affiliated Hospital of Soochow University. Before enrollment of first patient, the study protocol was approved by the Ethics Committee of all these research centers, and all patients provided written informed consent before recruitment. Shanghai Junshi Biomedical Technology Co., Ltd provides PD-1 antibody Toripalimab free of charge and purchases liability insurance for clinical trial subjects. This trial was initiated in December 2022 and is currently recruiting patients recruiting patients in all of the participating institutions above.

Study population

Inclusion in the TORCH-E trial requires that certain tumor and patient criteria are met. The inclusion and exclusion criteria are presented in Table 1.

Table 1 Inclusion and exclusion criteria

Inclusion criteria	Exclusion criteria
1. Age 18-70 years old, female and male	1. Pregnancy or breast-feeding women
2. Pathological confirmed adenocarcinoma	2. Pathological confirmed signet ring cell carcinoma
3. Clinical stage T1-3bN0, tumor maximum diameter less than 4cm	3. Clinical stage T1N0 and can be resected locally
	4. History of other malignancies within 5 years
4. The distance from anal verge less than 5 cm	5. Known history of severe neurological or mental illness
5. Without distance metastases	6. Serious medical illness, such as severe mental disorders, cardiac disease, uncontrolled infection, etc.
6. KPS >=70	
7. With good compliance	7. Immunodeficiency disease or long-term using of immunosuppressive agents
8. Microsatellite repair status is MSS/pMMR	
9. Without previous anti-cancer therapy or immunotherapy	8. Uncontrolled infection which needs systemic therapy
10. Signed the inform consent	9. Baseline blood and biochemical indicators do not meet the following criteria: neutrophils≥1.5×10^9/L, Hb≥90g/L, PLT≥100×10^9/L, ALT/AST ≤2.5 ULN, Cr≤ 1 ULN
	10. DPD deficiency
	11. Allergic to any component of the therapy

Treatment

Patients will first receive a total dose of 25Gy to the pelvic PTV delivered in fractions of 5 Gy. Almost two weeks after the last day of radiotherapy, four cycles of CAPOX chemotherapy and PD-1 antibody (Toripalimab) treatment will be performed. The CAPOX regimen consists of capecitabine and oxaliplatin, is repeated every three weeks. Oxaliplatin (130mg/m2) and Toripalimab (240 mg) will be administered intravenously on day 1. Capecitabine is administered orally at 1000 mg/m2 twice daily

For peer review only

for 14 consecutive days (3 weeks per cycle). After the last cycle neoadjuvant treatment, response evaluation is possible to performed. The multidisciplinary team will determine whether to proceed with local excision (TEM) or total mesorectal excision (TME) surgery based on the response evaluation. In cases where high-risk factors are identified after TEM, supplementary TME surgery is considered. Patients who achieve complete a CCR are eligible for the Watch & Wait strategy.

The treatment termination criteria will be as follows: (1) disease progression during treatment; (2) encounter intolerable toxicity; (3) need emergency surgical resection due to intestinal obstruction, intestinal perforation, intestinal bleeding, etc.; (4) request to withdraw from the cohort of this study due to various reasons; (5) accompanied by other non-tumor diseases make the patient unable to accept this treatment plan; (6) cannot complete the study plan due to various reasons.

Study endpoints

The primary endpoint is the CR rate, including rate of pCR and cCR. Specifically, it includes the following three situations: 1. ypT0 for patients with local excisions; 2. ypT0N0/pCR confirmed after TME surgery; 3. cCR assessment for Watch & Wait patients, conducted at neoadjuvant completion and sustained for a year. The cCR status is defined as undetectable tumor signs after neoadjuvant therapy through clinical examinations, including magnetic resonance imaging (MRI) of the pelvis, endoscopy, and digital rectal exam (DRE) according to the criteria set by Maas et al in 2011[25]. The definition of a cCR is (1) substantial downsizing with no residual tumor or residual fibrosis only (with low signal on high b-value DWI, if available). Residual wall thickening due to edema only was also an indication for a possible cCR; (2) no suspicious lymph nodes on MRI; (3) no residual tumor at endoscopy or only a small residual erythematous ulcer or scar; (4) negative biopsies from the scar, ulcer, or former tumor location; and (5) no palpable tumor, when initially palpable with digital rectal examination. If patients did not meet all of these criteria, they were regarded as incomplete responders. The pCR (ypT0) of LE specimen is designated by no visible tumor cells[8], and the pCR of TME specimen is defined as the absence of tumor cells at the primary site and regional lymph nodes. The secondary endpoints include the organ preservation rate, 3-year local recurrence free survival (LRFS) rate, 3-year disease free survival (DFS) rate**,** 3-year overall survival (OS) rate, grade 3-4 adverse effects rate, and patients' quality of life.

Assessment

Regular examinations at the following time points will be performed: baseline, before the second PD-1 monoclonal antibody, after the last cycle of neoadjuvant treatment but prior to surgery and at every visit during the follow-up. Patients will undergo response evaluation with MRI of the pelvis, computerized tomography (CT) of chest and abdomen, DRE, blood routine examination, liver and renal function tests, myocardial enzymogram, thyroid hormone, adrenal hormone, and serum tumor markers.

Radiological assessment will be according to the Response Evaluation Criteria in Solid Tumours (RECIST 1.1)[26] and immune Response Evaluation Criteria in Solid Tumours (iRECIST)[27]. The pathological evaluation is mainly used to evaluate the degree of tumour regression on the gross resected specimen. Pathological tumor regression grade (pTRG) will be evaluated according to the 8th American Joint Committee on Cancer (AJCC) Staging Manual by two independent pathologists.

Adverse effects will be assessed according to the Common Terminology Criteria for Adverse Event (CTCAE) v5.0, patients' quality of life (QoL) will be evaluated using the EORTC QLQ-C30 and

For peer review only

EORTC QLQ-CR29 scales. Additionally, the evaluation of anorectal and bowel function will be based on the Wexner score and LARS score[28, 29].

Sample size calculation and statistical analysis

This study is a prospective, single-arm, phase II clinical study. The primary endpoint is the CR rate. According to the previous reports, the reference CR rate is about 45% (null hypothesis, p0), and we assumed that the CR rate in this study can be increased to 70% (alternative hypothesis, p1). With the statistical power of 0.8 (β=0.2), the type I error rate of 0.05 (α= 0.05), approximately 30 patients will be required in this study. Considering the dropout rate of 10%, the sample size in this study will be 34 cases.

The primary endpoint of TORCH-E study is the CR rate, including rate of pCR and cCR. The OS will be calculated from the date of randomization to the date of death or the last follow- up. Survivals will be estimated by using the Kaplan-Meier method, including OS, DFS and LRFS. Endpoints related to the rate, including the cCR rate, pCR rate, organ preservation rate, toxicity occurrence, will be summarized with the frequency. The level of significance is $P < 0.05$. All data analyses were performed using SPSS 26.0.

Follow-up

The follow-up will be carried out every three months for at least three years postoperatively. The follow-up contents include the physical examination, tumor marker examination (CEA, CA199, AFP, CA724, CA242, CA50), and radiological examinations. MRI and CT scans will be performed alternately every three months. Colon endoscopy, late adverse effects collection, QoL assessment using EORTC questionnaires QLQ-C30 and QLQ-CR29, anorectal and bowel function using Wexner score and LARS score will be performed every 6 months. Colonoscopy, late adverse effects collection, QoL assessment (EORTC QLQ-C30/CR29), Wexner score and LARS score will be performed every 6 months. The events of local recurrence, distant metastasis and death will be recorded in detail.

Data collection, management and monitoring

The data collection and management will be achieved by researchers. The participants in the study and their personal data will be kept confidential. All participants will be assigned a unique number, all records will be affiliated with this number, and all study-related data will be anonymous. Finally, the clinical data will be analysed by the researchers. The results will be submitted for peer-reviewed publication and conference presentations.

Ethics and dissemination

This study was conducted in compliance with Declaration of Helsinki principles. Fudan University Shanghai Cancer Center (FUSCC) is the sponsor， who takes overall responsibility for the initiation and management of this trial. Additionally, FUSCC is in charge of study design, data collection, analysis, interpretation, and reporting, playing a role in ensuring ethical considerations, patient safety, and adherence to regulatory guidelines throughout the entire research process. All procedures involving human subjects were approved by the Ethics Committee of Fudan University Shanghai Cancer Center (Approval Number: 2209261-18). All patients signed informed consent forms before recruitment. The corresponding results will be disseminated via peer reviewed journals and conference presentations.

For peer review only

The participants in the study and their personal data during the study will be kept confidential in all study-related documents and publications.

Author affiliations

[1] Department of Radiation Oncology, Fudan University Shanghai Cancer Center, Shanghai, China.

[2] Department of Oncology, Shanghai Medical College, Fudan University, Shanghai, China.

[3] Department of Colorectal Surgery, Fudan University Shanghai Cancer Center, Shanghai, China.

Availability of data and materials

Trial results will be disseminated via peer reviewed scientific journals and conference presentations.

Acknowledgements

We would like to thank Shanghai Junshi Biomedical Technology Co., Ltd. for their drug support and having purchased liability insurance for clinical trial subjects.

Contributors

Prof. FX, ZZ, and XL designed the clinical study. YC, YW1 (Yaqi Wang), MZ, RY, WY, SZ and FX made substantial contributions to the organization of this study. HZ, JW, LS, YW2 (Yan Wang), XL, SC, ZZ and FX were responsible for patient recruitment. YC and FX contributed to the manuscript drafting and revising. All authors gave final approval the version of the protocol, and it was also approved by local researchers of the participating centers.

Funding

This study was supported by Shanghai Junshi Biomedical Technology Co., Ltd. (Project protocol No. JS001-ISS-C0402), the National Natural Science Foundation of China (Grant No. 82102978) and the National Key R&D Program of China (Grant No.2022YFC2503700, 2022YFC2503702).

Competing interests

The authors declare that they have no competing interests.

Patient consent for publication

Consent obtained directly from patient(s).

References

1. Wiltink LM, Marijnen CA, Meershoek-Klein Kranenbarg E, van de Velde CJ, Nout RA: A comprehensive longitudinal overview of health-related quality of life and symptoms after treatment for rectal cancer in the TME trial. *Acta Oncol* 2016, 55(4):502-508.

2. van der Valk MJM, Hilling DE, Bastiaannet E, Meershoek-Klein Kranenbarg E, Beets GL, Figueiredo NL, Habr-Gama A, Perez RO, Renehan AG, van de Velde CJH *et al*: Long-term outcomes

For peer review only

of clinical complete responders after neoadjuvant treatment for rectal cancer in the International Watch & Wait Database (IWWD): an international multicentre registry study. *Lancet* 2018, 391(10139):2537-2545.

3. Bahadoer RR, Dijkstra EA, van Etten B, Marijnen CAM, Putter H, Kranenbarg EM, Roodvoets AGH, Nagtegaal ID, Beets-Tan RGH, Blomqvist LK *et al*: Short-course radiotherapy followed by chemotherapy before total mesorectal excision (TME) versus preoperative chemoradiotherapy, TME, and optional adjuvant chemotherapy in locally advanced rectal cancer (RAPIDO): a randomised, open-label, phase 3 trial. *Lancet Oncol* 2021, 22(1):29-42.

4. Conroy T, Bosset JF, Etienne PL, Rio E, Francois E, Mesgouez-Nebout N, Vendrely V, Artignan X, Bouche O, Gargot D *et al*: Neoadjuvant chemotherapy with FOLFIRINOX and preoperative chemoradiotherapy for patients with locally advanced rectal cancer (UNICANCER-PRODIGE 23): a multicentre, randomised, open-label, phase 3 trial. *Lancet Oncol* 2021, 22(5):702-715.

5. Borschitz T, Wachtlin D, Mohler M, Schmidberger H, Junginger T: Neoadjuvant chemoradiation and local excision for T2-3 rectal cancer. *Ann Surg Oncol* 2008, 15(3):712-720.

6. Nair RM, Siegel EM, Chen DT, Fulp WJ, Yeatman TJ, Malafa MP, Marcet J, Shibata D: Long-term results of transanal excision after neoadjuvant chemoradiation for T2 and T3 adenocarcinomas of the rectum. *J Gastrointest Surg* 2008, 12(10):1797-1805; discussion 1805-1796.

7. Garcia-Aguilar J, Renfro LA, Chow OS, Shi Q, Carrero XW, Lynn PB, Thomas CR, Jr., Chan E, Cataldo PA, Marcet JE *et al*: Organ preservation for clinical T2N0 distal rectal cancer using neoadjuvant chemoradiotherapy and local excision (ACOSOG Z6041): results of an open-label, single-arm, multi-institutional, phase 2 trial. *Lancet Oncol* 2015, 16(15):1537-1546.

8. Serra-Aracil X, Pericay C, Badia-Closa J, Golda T, Biondo S, Hernandez P, Targarona E, Borda-Arrizabalaga N, Reina A, Delgado S *et al*: Short-term outcomes of chemoradiotherapy and local excision versus total mesorectal excision in T2-T3ab,N0,M0 rectal cancer: a multicentre randomised, controlled, phase III trial (the TAU-TEM study). *Ann Oncol* 2023, 34(1):78-90.

9. Simon Parkinson Bach, Johannes de Wilt, Femke Peters, Karen-Lise Garm Spindler, Ane Appelt, Mark Teo, Victoria Homer, Natalie Abbott, lan Geh, Stephan Korsgen *et al*: Can we save the rectum by watchful waiting or transanal surgery following (chemo)Radiotherapy versus total mesorectal excision for early rectal cancer? In: *ASCO, 2022.* Chicago.

10. Gerard JP, Barbet N, Schiappa R, Magne N, Martel I, Mineur L, Deberne M, Zilli T, Dhadda A, Myint AS *et al*: Neoadjuvant chemoradiotherapy with radiation dose escalation with contact x-ray brachytherapy boost or external beam radiotherapy boost for organ preservation in early cT2-cT3 rectal adenocarcinoma (OPERA): a phase 3, randomised controlled trial. *Lancet Gastroenterol Hepatol* 2023.

11. Jensen LH: Curative chemoradiation for low rectal cancer: primary clinical outcomes from a multicenter phase II trial. In: *ASCO, 2022.* Chicago.

12. Dovedi SJ, Illidge TM: The antitumor immune response generated by fractionated radiation therapy may be limited by tumor cell adaptive resistance and can be circumvented by PD-L1 blockade. *Oncoimmunology* 2015, 4(7):e1016709.

13. Dovedi SJ, Adlard AL, Lipowska-Bhalla G, McKenna C, Jones S, Cheadle EJ, Stratford IJ, Poon E, Morrow M, Stewart R *et al*: Acquired resistance to fractionated radiotherapy can be overcome by concurrent PD-L1 blockade. *Cancer research* 2014, 74(19):5458-5468.

14. Zhou L, Yu G, Y S: The clinical efficacy and safety of neoadjuvant chemoradiation therapy with immunotherapy for the organ preservation of ultra low rectal cancer: A single arm and open label exploratory study. In: *ASCO, 2022.* Chicago.

For peer review only

15. Jin J, Tang Y, Hu C, Jiang LM, Jiang J, Li N, Liu WY, Chen SL, Li S, Lu NN *et al*: Multicenter, Randomized, Phase III Trial of Short-Term Radiotherapy Plus Chemotherapy Versus Long-Term Chemoradiotherapy in Locally Advanced Rectal Cancer (STELLAR). *J Clin Oncol* 2022, 40(15):1681-1692.

16. Bujko K, Wyrwicz L, Rutkowski A, Malinowska M, Pietrzak L, Krynski J, Michalski W, Oledzki J, Kusnierz J, Zajac L *et al*: Long-course oxaliplatin-based preoperative chemoradiation versus 5 x 5 Gy and consolidation chemotherapy for cT4 or fixed cT3 rectal cancer: results of a randomized phase III study. *Ann Oncol* 2016, 27(5):834-842.

17. Crocenzi T, Cottam B, Newell P, Wolf RF, Hansen PD, Hammill C, Solhjem MC, To YY, Greathouse A, Tormoen G *et al*: A hypofractionated radiation regimen avoids the lymphopenia associated with neoadjuvant chemoradiation therapy of borderline resectable and locally advanced pancreatic adenocarcinoma. *J Immunother Cancer* 2016, 4:45.

18. Lan J, Li R, Yin LM, Deng L, Gui J, Chen BQ, Zhou L, Meng MB, Huang QR, Mo XM *et al*: Targeting Myeloid-derived Suppressor Cells and Programmed Death Ligand 1 Confers Therapeutic Advantage of Ablative Hypofractionated Radiation Therapy Compared With Conventional Fractionated Radiation Therapy. *Int J Radiat Oncol Biol Phys* 2018, 101(1):74-87.

19. Dewan MZ, Galloway AE, Kawashima N, Dewyngaert JK, Babb JS, Formenti SC, Demaria S: Fractionated but not single-dose radiotherapy induces an immune-mediated abscopal effect when combined with anti-CTLA-4 antibody. *Clin Cancer Res* 2009, 15(17):5379-5388.

20. Wang Y, Shen L, Wan J, Zhang H, Wu R, Wang J, Wang Y, Xu Y, Cai S, Zhang Z *et al*: Short-course radiotherapy combined with CAPOX and Toripalimab for the total neoadjuvant therapy of locally advanced rectal cancer: a randomized, prospective, multicentre, double-arm, phase II trial (TORCH). *BMC Cancer* 2022, 22(1):274.

21. Wang Y, Xia F, Shen L, Wan J, Zhang H, Wu R, Wang J, Wang Y, Xu Y, Cai S *et al*: Short-course radiotherapy combined with capox and toripalimab for the total neoadjuvant therapy of locally advanced rectal cancer: Preliminary findings from a randomized, prospective, multicenter, double-arm, phase ii trial (Torch). In: *ASCO, 2022.* Chicago.

22. Wang Y, Shen L, Wan J, Zhang H, Wu R, Wang J, Wang Y, Xu Y, Cai S, Zhang Z *et al*: Neoadjuvant chemoradiotherapy combined with immunotherapy for locally advanced rectal cancer: A new era for anal preservation. *Front Immunol* 2022, 13:1067036.

23. Shamseddine A, Zeidan YH, El Husseini Z, Kreidieh M, Al Darazi M, Turfa R, Kattan J, Khalifeh I, Mukherji D, Temraz S *et al*: Efficacy and safety-in analysis of short-course radiation followed by mFOLFOX-6 plus avelumab for locally advanced rectal adenocarcinoma. *Radiat Oncol* 2020, 15(1):233.

24. Sheng Dai, Fei Wang, Yanbin Shen, Liming Shi, Lingna Xu, Chuanxi Lai, Feixiang Zhang, Kangke Zhang, Dengyong Xu, Lina Shan *et al*: Efficacy and safety of neoadjuvant preoperative short-course radiation followed by envafolimab plus CAPEOX in microsatellite stable (MSS)/mismatch repair proficient (pMMR) locally advanced rectal cancer. In: *2023 ASCO Gastrointestinal Cancers Symposium.* San Francisco.

25. Maas M, Beets-Tan RG, Lambregts DM, Lammering G, Nelemans PJ, Engelen SM, van Dam RM, Jansen RL, Sosef M, Leijtens JW *et al*: Wait-and-see policy for clinical complete responders after chemoradiation for rectal cancer. *J Clin Oncol* 2011, 29(35):4633-4640.

26. Eisenhauer EA, Therasse P, Bogaerts J, Schwartz LH, Sargent D, Ford R, Dancey J, Arbuck S, Gwyther S, Mooney M *et al*: New response evaluation criteria in solid tumours: revised RECIST

For peer review only

guideline (version 1.1). *Eur J Cancer* 2009, 45(2):228-247.
27. Seymour L, Bogaerts J, Perrone A, Ford R, Schwartz LH, Mandrekar S, Lin NU, Litiere S, Dancey J, Chen A *et al*: iRECIST: guidelines for response criteria for use in trials testing immunotherapeutics. *Lancet Oncol* 2017, 18(3):e143-e152.
28. Jorge JM, Wexner SD: Etiology and management of fecal incontinence. *Dis Colon Rectum* 1993, 36(1):77-97.
29. Emmertsen KJ, Laurberg S: Low anterior resection syndrome score: development and validation of a symptom-based scoring system for bowel dysfunction after low anterior resection for rectal cancer. *Ann Surg* 2012, 255(5):922-928.

Figure Legends

Figure.1 Flow chart of the TORCH-E study. Note: SCRT, short- course radiotherapy; mAb, monoclonal antibody; CAPOX, capecitabine plus oxaliplatin; cCR: clinical complete response; CR: clinical response; TME: total mesorectal excision.

For peer review only

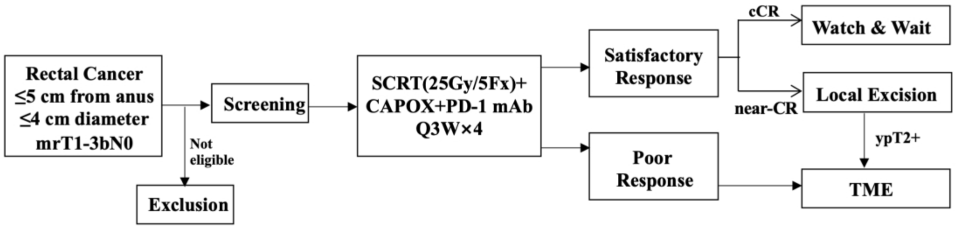

Flow chart of the TORCH-E study.

338x190mm (150 x 150 DPI)

STANDARD PROTOCOL ITEMS: RECOMMENDATIONS FOR INTERVENTIONAL TRIALS

SPIRIT 2013 Checklist: Recommended items to address in a clinical trial protocol and related documents*

Section/item	Item No	Description	Addressed on page number
**Administrative information**			
Title	1	Descriptive title identifying the study design, population, interventions, and, if applicable, trial acronym	Page1
Trial registration	2a	Trial identifier and registry name. If not yet registered, name of intended registry	Page2
	2b	All items from the World Health Organization Trial Registration Data Set	NA
Protocol version	3	Date and version identifier	Page4
Funding	4	Sources and types of financial, material, and other support	Page8
Roles and responsibilities	5a	Names, affiliations, and roles of protocol contributors	Page7-8
	5b	Name and contact information for the trial sponsor	Page1
	5c	Role of study sponsor and funders, if any, in study design; collection, management, analysis, and interpretation of data; writing of the report; and the decision to submit the report for publication, including whether they will have ultimate authority over any of these activities	Page8
	5d	Composition, roles, and responsibilities of the coordinating centre, steering committee, endpoint adjudication committee, data management team, and other individuals or groups overseeing the trial, if applicable (see Item 21a for data monitoring committee)	NA

For peer review only - http://bmjopen.bmj.com/site/about/guidelines.xhtml

Introduction

Background and rationale	6a	Description of research question and justification for undertaking the trial, including summary of relevant studies (published and unpublished) examining benefits and harms for each intervention	Page3-4
	6b	Explanation for choice of comparators	Page4
Objectives	7	Specific objectives or hypotheses	Page4
Trial design	8	Description of trial design including type of trial (eg, parallel group, crossover, factorial, single group), allocation ratio, and framework (eg, superiority, equivalence, noninferiority, exploratory)	Page4

Methods: Participants, interventions, and outcomes

Study setting	9	Description of study settings (eg, community clinic, academic hospital) and list of countries where data will be collected. Reference to where list of study sites can be obtained	Page5
Eligibility criteria	10	Inclusion and exclusion criteria for participants. If applicable, eligibility criteria for study centres and individuals who will perform the interventions (eg, surgeons, psychotherapists)	Page5
Interventions	11a	Interventions for each group with sufficient detail to allow replication, including how and when they will be administered	Page5
	11b	Criteria for discontinuing or modifying allocated interventions for a given trial participant (eg, drug dose change in response to harms, participant request, or improving/worsening disease)	NA
	11c	Strategies to improve adherence to intervention protocols, and any procedures for monitoring adherence (eg, drug tablet return, laboratory tests)	NA
	11d	Relevant concomitant care and interventions that are permitted or prohibited during the trial	Page5
Outcomes	12	Primary, secondary, and other outcomes, including the specific measurement variable (eg, systolic blood pressure), analysis metric (eg, change from baseline, final value, time to event), method of aggregation (eg, median, proportion), and time point for each outcome. Explanation of the clinical relevance of chosen efficacy and harm outcomes is strongly recommended	Page6
Participant timeline	13	Time schedule of enrolment, interventions (including any run-ins and washouts), assessments, and visits for participants. A schematic diagram is highly recommended (see Figure)	Figure1

For peer review only - http://bmjopen.bmj.com/site/about/guidelines.xhtml

Sample size	14	Estimated number of participants needed to achieve study objectives and how it was determined, including clinical and statistical assumptions supporting any sample size calculations	Page6-7
Recruitment	15	Strategies for achieving adequate participant enrolment to reach target sample size	NA

Methods: Assignment of interventions (for controlled trials)

Allocation:

Sequence generation	16a	Method of generating the allocation sequence (eg, computer-generated random numbers), and list of any factors for stratification. To reduce predictability of a random sequence, details of any planned restriction (eg, blocking) should be provided in a separate document that is unavailable to those who enrol participants or assign interventions	NA
Allocation concealment mechanism	16b	Mechanism of implementing the allocation sequence (eg, central telephone; sequentially numbered, opaque, sealed envelopes), describing any steps to conceal the sequence until interventions are assigned	NA
Implementation	16c	Who will generate the allocation sequence, who will enrol participants, and who will assign participants to interventions	Prof. Fan Xia
Blinding (masking)	17a	Who will be blinded after assignment to interventions (eg, trial participants, care providers, outcome assessors, data analysts), and how	NA
	17b	If blinded, circumstances under which unblinding is permissible, and procedure for revealing a participant's allocated intervention during the trial	NA

Methods: Data collection, management, and analysis

Data collection methods	18a	Plans for assessment and collection of outcome, baseline, and other trial data, including any related processes to promote data quality (eg, duplicate measurements, training of assessors) and a description of study instruments (eg, questionnaires, laboratory tests) along with their reliability and validity, if known. Reference to where data collection forms can be found, if not in the protocol	Page7
	18b	Plans to promote participant retention and complete follow-up, including list of any outcome data to be collected for participants who discontinue or deviate from intervention protocols	Page7

Data management	19	Plans for data entry, coding, security, and storage, including any related processes to promote data quality (eg, double data entry; range checks for data values). Reference to where details of data management procedures can be found, if not in the protocol	NA
Statistical methods	20a	Statistical methods for analysing primary and secondary outcomes. Reference to where other details of the statistical analysis plan can be found, if not in the protocol	Page 6-7
	20b	Methods for any additional analyses (eg, subgroup and adjusted analyses)	Page 6-7
	20c	Definition of analysis population relating to protocol non-adherence (eg, as randomised analysis), and any statistical methods to handle missing data (eg, multiple imputation)	NA
**Methods: Monitoring**			
Data monitoring	21a	Composition of data monitoring committee (DMC); summary of its role and reporting structure; statement of whether it is independent from the sponsor and competing interests; and reference to where further details about its charter can be found, if not in the protocol. Alternatively, an explanation of why a DMC is not needed	NA
	21b	Description of any interim analyses and stopping guidelines, including who will have access to these interim results and make the final decision to terminate the trial	Page5-6
Harms	22	Plans for collecting, assessing, reporting, and managing solicited and spontaneously reported adverse events and other unintended effects of trial interventions or trial conduct	Page6
Auditing	23	Frequency and procedures for auditing trial conduct, if any, and whether the process will be independent from investigators and the sponsor	NA
**Ethics and dissemination**			
Research ethics approval	24	Plans for seeking research ethics committee/institutional review board (REC/IRB) approval	Page7
Protocol amendments	25	Plans for communicating important protocol modifications (eg, changes to eligibility criteria, outcomes, analyses) to relevant parties (eg, investigators, REC/IRBs, trial participants, trial registries, journals, regulators)	NA

For peer review only - http://bmjopen.bmj.com/site/about/guidelines.xhtml

Consent or assent	26a	Who will obtain informed consent or assent from potential trial participants or authorised surrogates, and how (see Item 32)	NA
	26b	Additional consent provisions for collection and use of participant data and biological specimens in ancillary studies, if applicable	NA
Confidentiality	27	How personal information about potential and enrolled participants will be collected, shared, and maintained in order to protect confidentiality before, during, and after the trial	Page7
Declaration of interests	28	Financial and other competing interests for principal investigators for the overall trial and each study site	Page8
Access to data	29	Statement of who will have access to the final trial dataset, and disclosure of contractual agreements that limit such access for investigators	NA
Ancillary and post-trial care	30	Provisions, if any, for ancillary and post-trial care, and for compensation to those who suffer harm from trial participation	NA
Dissemination policy	31a	Plans for investigators and sponsor to communicate trial results to participants, healthcare professionals, the public, and other relevant groups (eg, via publication, reporting in results databases, or other data sharing arrangements), including any publication restrictions	Page8
	31b	Authorship eligibility guidelines and any intended use of professional writers	NA
	31c	Plans, if any, for granting public access to the full protocol, participant-level dataset, and statistical code	NA
**Appendices**			
Informed consent materials	32	Model consent form and other related documentation given to participants and authorised surrogates	Supplementary material
Biological specimens	33	Plans for collection, laboratory evaluation, and storage of biological specimens for genetic or molecular analysis in the current trial and for future use in ancillary studies, if applicable	NA

*It is strongly recommended that this checklist be read in conjunction with the SPIRIT 2013 Explanation & Elaboration for important clarification on the items. Amendments to the protocol should be tracked and dated. The SPIRIT checklist is copyrighted by the SPIRIT Group under the Creative Commons "Attribution-NonCommercial-NoDerivs 3.0 Unported" license.
